# Co-Occurrence of Anti-Synthetase Syndrome and Sjögren Disease: A Case-Based Review

**DOI:** 10.3390/jcm14155395

**Published:** 2025-07-31

**Authors:** Andrea Pilato, Giorgio D’Avanzo, Francesca Di Nunzio, Annalisa Marino, Alessia Gallo, Irene Genovali, Letizia Pia Di Corcia, Chiara Taffon, Giuseppe Perrone, Vasiliki Liakouli, Luca Navarini, Roberto Giacomelli, Onorina Berardicurti, Raffaele Antonelli Incalzi

**Affiliations:** 1 Clinical Unit of Immunorheumatology, Fondazione Policlinico Universitario Campus Bio-Medico, Via Alvaro del Portillo, 200, 00128 Roma, Italy; andrea.pilato@unicampus.it (A.P.); a.marino@policlinicocampus.it (A.M.); irene.genovali@policlinicocampus.it (I.G.); letiziapia.dicorcia@unicampus.it (L.P.D.C.); l.navarini@policlinicocampus.it (L.N.); r.giacomelli@policlinicocampus.it (R.G.); o.berardicurti@policlinicocampus.it (O.B.); 2 Research Unit of Immunorheumatology, Department of Medicine and Surgery, Università Campus Bio-Medico di Roma, Via Alvaro del Portillo, 200, 00128 Roma, Italy; 3 Diagnostic and Therapeutic Medicine Department, Fondazione Policlinico Universitario Campus Bio-Medico, 00128 Rome, Italy; giorgio.davanzo@policlinicocampus.it (G.D.); francesca.dinunzio@policlinicocampus.it (F.D.N.); alessia.gallo@unicampus.it (A.G.); 4 Anatomical Pathology Operative Research Unit, Fondazione Policlinico Universitario Campus Bio-Medico, 00128 Rome, Italy; c.taffon@policlinicocampus.it (C.T.); g.perrone@policlinicocampus.it (G.P.); 5 Research Unit of Anatomical Pathology, Department of Medicine and Surgery, Università Campus Bio-Medico di Roma, 00128 Rome, Italy; 6 Rheumatology Unit, Department of Precision Medicine, University of Campania L. Vanvitelli, 81100 Caserta, Italy; 7 Research Unit of Geriatrics, Department of Medicine and Surgery, Università Campus Bio-Medico di Roma, 00128 Rome, Italy; r.antonelli@policlinicocampus.it; 8 Operative Research Unit of Internal Medicine, Fondazione Policlinico Universitario Campus Bio-Medico, 00128 Rome, Italy

**Keywords:** anti-synthetase syndrome, Sjögren’s disease, idiopathic inflammatory myopathy, interstitial lung disease

## Abstract

**Background**: Anti-synthetase Syndrome (ASyS) is an idiopathic inflammatory myopathy characterized by muscle weakness and inflammatory infiltrates in muscles. Sjogren’s disease (SD) is an autoimmune condition primarily affecting exocrine glands. Both these conditions may present lung involvement. We describe a female patient with anti-synthetase/SD overlap syndrome and review the literature to identify published cases describing this overlap, aiming to better define its clinical, radiological, and serological features. **Methods**: The case description was based on a retrospective collection of clinical, laboratory, and imaging data related to the patient’s diagnostic process and clinical course. Data were anonymized and handled in accordance with the competent territorial Ethics Committee. A literature review was performed using the MEDLINE and Scopus databases by combining the keywords “Anti-Synthetase syndrome”, “Sjögren disease”, “Sjögren syndrome”, “Myositis”, and “Interstitial lung disease” (ILD). Published cases were selected if they met the 2016 EULAR/ACR criteria for SD and at least one of the currently proposed classification criteria for ASyS. **Results**: The described case concerns a 68-year-old woman with rapidly progressive ILD. The diagnosis of anti-synthetase/SD overlap syndrome was based on clinical, serological (anti-Ro52 and anti-PL7 antibodies), histological, and radiological findings. Despite immunosuppressive and antifibrotic treatment, the clinical course worsened, leading to a poor outcome. In addition, six relevant cases were identified in the literature. Clinical presentations, autoantibody profiles, radiological findings, and outcomes were highly heterogeneous. Among the reported cases, no standardized treatment protocols were adopted, reflecting the lack of consensus in managing this rare condition. **Conclusions**: In anti-synthetase/SD overlap syndrome, ILD may follow a rapidly progressive course. Early recognition can be challenging, especially in the absence of muscular involvement. This case-based review highlights the need for more standardized approaches to the diagnosis and management of this rare and complex overlap syndrome.

## 1. Introduction

Anti-synthetase Syndrome (ASyS) is a rare autoimmune disorder characterized by the presence of specific autoantibodies targeting aminoacyl-tRNA synthetases. According to Orphanet, the global prevalence of ASyS is estimated as 1-9/10,000; however, the real incidence remains not fully defined [1]. ASyS is typically characterized by interstitial lung disease (ILD), myositis, non-erosive arthritis, Raynaud’s phenomenon (RP), mechanic’s hands, and fever episodes, without apparent secondary causes [2]. ASyS is part of a broader and heterogeneous group of conditions known as idiopathic inflammatory myopathies (IIMs), sharing clinical features of progressive muscle weakness and the presence of inflammatory infiltrates in muscles. IIMs may be present within the context of another systemic rheumatic condition, such as systemic lupus erythematosus (SLE), systemic sclerosis (SSc), mixed connective tissue disease (MCTD), and, less frequently, rheumatoid arthritis (RA) and Sjogren’s disease (SD) [3,4]. As far as SD is considered, it is a chronic systemic autoimmune disease characterized by the lymphocytic infiltration of the exocrine glands and B-cell dysfunction. The most specific autoantibody associated with this autoimmune condition is anti-Ro/SSA. The clinical picture is characterized by sicca syndrome, resulting from the lymphocytic infiltration of the glands, which is frequently associated with fatigue, musculoskeletal pain, and a wide range of systemic manifestations including nephritis, cytopenia, ILD, vasculitis, peripheral neuropathy, myelopathy, and cognitive disruptions. Additionally, lymphoma occurs as a complication in around 2–5% of patients [5].

The recent identification of myositis-specific antibodies has led to the recognition of a rare subset of patients with SD who also fulfill the criteria for ASyS [6]. Such overlap syndromes may present with complex and atypical clinical and serological features, highlighting the importance of comprehensive immunological screening.

This case-based review presents a rare instance of anti-synthetase/SD overlap syndrome and includes a structured analysis of the existing literature to identify all published cases describing this rare association, with the aim of better characterizing its clinical, radiological, and serological features and promoting its early recognition.

## 2. Methods

The case description was based on a retrospective collection of anonymized clinical, laboratory, and radiological data, in accordance with approval from the local Ethics Committee.

For the literature review, a structured search was conducted using the MEDLINE (via PubMed) and Scopus databases. The search included articles published between 1 January 2000, and 30 April 2025. The following search terms were used in combination: “Anti-Synthetase syndrome”, “Sjögren disease”, “Sjögren syndrome”, “Myositis”, and “Interstitial lung disease”. Boolean operators (AND, OR) were applied to optimize the search strategy. Eligible publications included case reports, case series, cohort studies, and letters to the editor, provided that individual cases were described with sufficient clinical detail. The inclusion criteria were (1) adult patients (>18 years) with a diagnosis of both ASyS and SD; (2) the fulfillment of the 2016 ACR/EULAR classification criteria for SD; and (3) the fulfillment of at least one of the proposed classification criteria for ASyS (Solomon et al., Connors et al., or Lega et al.) [7,8,9,10,11]. Studies not written in English, conference abstracts without the full text, and publications lacking sufficient clinical data to verify the diagnosis of the overlap syndrome were excluded. Titles and abstracts were screened independently by two reviewers, and the full texts of eligible articles were assessed for inclusion.

## 3. Case Presentation

**Figure 1:**
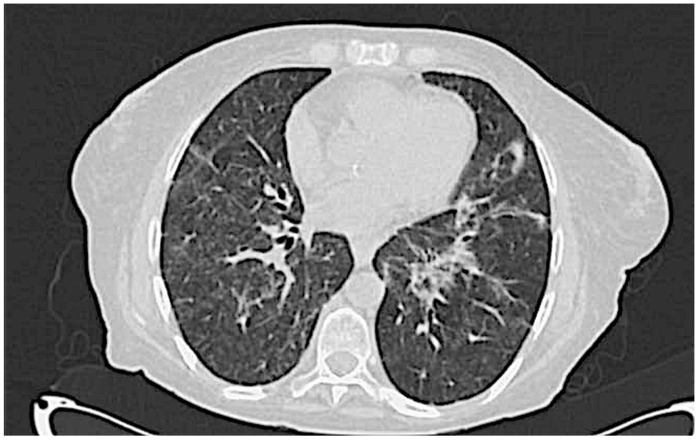
First HRCT scan showing interstitial involvement characterized by widespread thickening of inter- and intra-lobular septa centrally and peripherally in both lungs, along the pleural surfaces and fissural planes. This is accompanied by multiple areas of increased density with a ground-glass appearance.

A 68-year-old Caucasian woman was referred to our center in April 2023 for an evaluation of a persistent fever, xerostomia, myalgia, and worsening dyspnea. She denied unintentional weight loss, photosensitivity, alopecia, oral ulcers, recurrent serositis, xeropthalmia, and Raynaud Phenomenon (RP). A physical examination revealed bilateral fine crackles, without muscles weakness, rash, arthritis, or lymphadenopathies. The patient had no significant past medical history and reported no tobacco use, no relevant occupational or environmental exposures, and no contact with pets, birds, or other animals at risk. Five months earlier (January 2023), she had been admitted to another center for respiratory symptoms. High-resolution computed tomography (HRCT) findings at that time (Figure 1) were consistent with interstitial lung disease (ILD), and infectious and neoplastic causes were excluded. Blood tests showed antinuclear antibodies (ANA) 1:1280 with an anti-Golgi pattern, extractable nuclear antigen (ENA) Ro/SSA (Ro-52) 49 UI/mL, and a normal level of muscle enzymes: creatine kinase (CK) 11 UI/L, myoglobin 47 ng/mL, and glutamate oxaloacetate transaminase (GOT) 28 U/L. Pulmonary function tests (PFTs) showed normal volumes and a moderately reduced diffusing capacity for carbon monoxide (DLCO): vital capacity (VC) 1.96 L, forced vital capacity (FVC) 1.96 L, forced expiratory volume in one second (FEV1) 1.52 L, FEV1/FVC 77.44%, total lung capacity (TLC) 83%, alveolar volume (Va) 3.64 L, and TLCO (Va) 46%. At that time, an autoimmune etiology was considered among the differential diagnoses; however, no definitive classification was reached.

**Figure 2:**
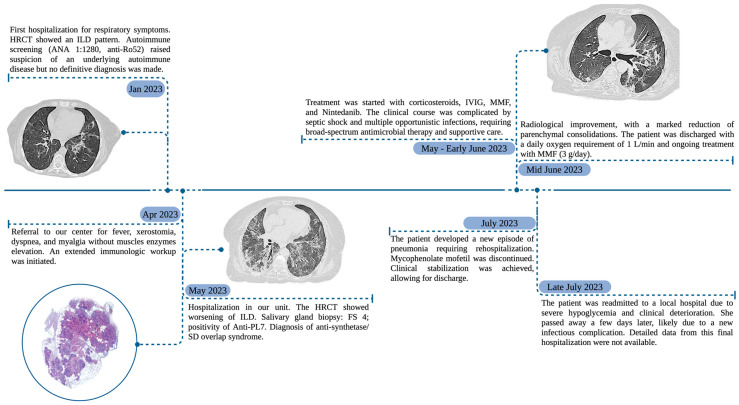
Timeline summarizing the clinical course of the patient from the first hospitalization in January 2023 to the final outcome in July 2023. Abbreviation: HRCT, high-resolution computed tomography; ILD, interstitial lung disease; FS, focus score; SD, Sjögren disease; IVIG, intravenous immunoglobulin; MMF, mycophenolate mofetil.

A timeline summarizing the clinical, diagnostic, and therapeutic courses of the patient is provided in Figure 2.

**Figure 3:**
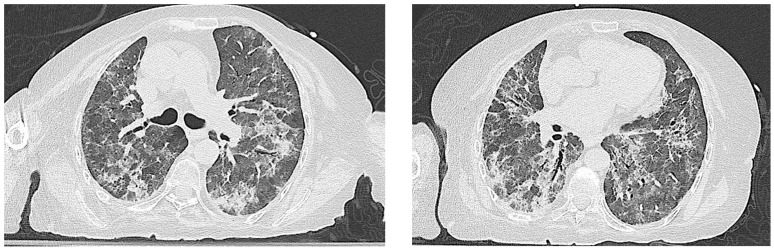
Progression of parenchymal damage, characterized by the expansion of ground-glass opacities and the inflammatory superimposition of interstitial pathology.

The patient’s clinical condition gradually worsened, leading to hospital admission to our unit in May 2023. A new HRCT scan showed the progression of ILD with increased ground-glass opacities (Figure 3). The arterial blood gas analysis on a high flow nasal cannula (70% FiO_2_) showed a P/F ratio of 137. Inflammatory markers were elevated (CRP 12.92 mg/dL; procalcitonin 1.11 ng/mL) with normal liver and kidney function. Bronchoalveolar lavage (BAL) showed neutrophilic inflammation and an elevated CD4/CD8 ratio. Infectious and neoplastic screenings were negative.

**Figure 4:**
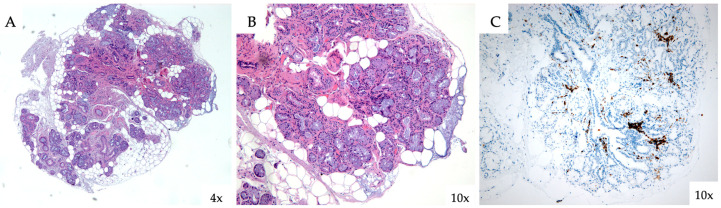
Labial minor salivary glands were inspected using light microscopy. In images (**A**,**B**), the histological preparation is stained with hematoxylin–eosin, while image (**C**) shows immunohisto-chemistry for CD3.

Given the autoimmune serology, further workup including a salivary gland biopsy confirmed focal lymphocytic sialadenitis (focus score of 4), consistent with SD (Figure 4). Anti PL-7 antibodies were then documented using the immunoblotting method, with strong positivity, leading to the diagnosis of anti-synthetase/SD overlap syndrome. The diagnosis of anti-synthetase syndrome was supported by both the Connors et al. classification criteria and the preliminary criteria proposed by the CLASS Project [9,12,13].

Treatment with methylprednisolone bolus (1 g/day for 3 days and subsequently tapered to a dosage of 20 mg/day), intravenous immunoglobulin (IVIG, 400 mg per kg per day for 5 days), mycophenolate mofetil (up to 3 g/day), and Nintedanib (later discontinued due to the occurrence of diarrhea) was started.

The course was complicated by septic shock (K. pneumoniae CRE), treated successfully with meropenem/vaborbactam and amikacin. Additional opportunistic infections (CMV, HSV, *Candida* spp.) required antiviral and antifungal therapies. Stomatitis and generalized edema occurred, managed with parenteral nutrition and diuretics.

**Figure 5:**
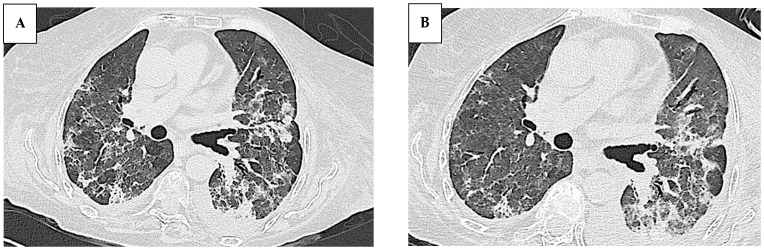

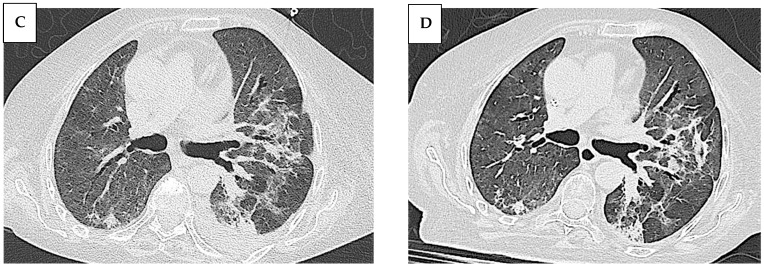
Alteration of radiologic ILD features due to pulmonary infections. Serial HRCT scan showing a reduction in areas of parenchymal consolidation before (**A**), during (**B**,**C**), and after (**D**) the immunosuppressive, antibacterial, antiviral, and antifungal therapies.

Gradually, clinical and radiological improvements were observed. Serial HRCT scans showed a marked reduction in the areas of parenchymal consolidation, although the fibrotic components of the interlobular septa were persistent (Figure 5). The patient was then discharged home in June 2023, with an oxygen requirement of 1 L/minute daily and 3 g/day of mycophenolate mofetil, with the stability of the clinical condition.

A second IVIG course was administered five weeks later; a chest HRCT scan showed the partial remission of parenchymal fibrotic involvement. Pulmonary function tests were not conducted during the follow-up due to the patient’s inability to complete the examination.

In July 2023, a new development of pneumonia required rehospitalization. Mycophenolate mofetil was discontinued and replaced with methylprednisolone at a dosage of 1 mg/kg; antibiotics treatment led to stabilization and the patient was then discharged in a stable clinical condition. However, she was readmitted shortly afterward to a local hospital for logistical reasons, due to severe hypoglycemia and clinical deterioration. She died a few days later, likely due to a new infectious episode. Unfortunately, detailed clinical data from this final hospitalization were not available.

## 4. Literature Review

Few cases of anti-synthetase/SD overlap syndromes have been described in the scientific literature so far, mostly in association with other connective tissue diseases (Table 1). The first two cases were identified in a multicenter cohort study of 1320 individuals with primary SD and IIMs published by Colafrancesco S. et al. in 2015 [6]. The first patient was a 37-year-old woman with proximal muscle weakness, elevated serum creatine kinase levels (18× upper limit), and interstitial pneumonia. Laboratory testing revealed positivity for ANA, anti-SSA, anti-SSB, anti-RNP, and anti-Jo1 antibodies. Electromyography confirmed myopathic changes and a muscle biopsy showed a polymyositis-like inflammatory pattern. After the failure of first-line immunosuppressive therapies including cyclosporine and mycophenolate mofetil, she was treated with rituximab (five cycles of 1 g two weeks apart every six months), achieving complete clinical remission.

The second patient was a 53-year-old man with ILD and proximal muscle weakness who tested positive for ANA, anti-SSA, and anti-Jo1 antibodies. His creatine kinase levels were markedly elevated (40× upper limit), and both an electromyography and muscle biopsy confirmed a polymyositis-like pattern. He initially received steroid pulses with a partial benefit, followed by mycophenolate mofetil (2 g/day); his clinical outcome was still under evaluation at the time of publication. In the same study, a third SD patient tested positive for anti-JO1 antibodies without fulfilling the ASyS criteria at the time of the evaluation. The authors emphasized the risk of disease progression over time.

In 2011, Hervier B. et al. [14] described a case of a 21-year-old African woman who presented with anti-OJ-positive ASyS overlapping with SSc and SD. Over two months, she developed progressive dyspnea, muscle weakness, and ILD with a honeycomb pattern on a chest HRCT scan. Severe muscular involvement was confirmed by an electromyography and muscle biopsy. The patient was initially treated with steroids at a dose of 1 mg/kg per day and monthly pulses of cyclophosphamide (CYC) at a dosage of 0.7 g/m^2^. After receiving six pulses of CYC, it was replaced by azathioprine (AZA) at a dose of 2 mg/kg per day. Over 41 months of follow-up, the patient improved in their muscular recovery and pulmonary function. However, she also developed additional scleroderma-related symptoms such as sclerodactyly, esophageal issues, and recurrent digital ulcers, which required further management [14].

In August 2020, Yoshikawa M. et al. [15] described an overlapping syndrome of IgG4-related autoimmune pancreatitis, SD, and ASyS. The patient was a 55-year-old man initially diagnosed with autoimmune pancreatitis, later developing finger swelling, stiffness, and ILD. SD was diagnosed based on a minor salivary gland biopsy and positive anti-Ro/SSA antibodies. At age 58, he was hospitalized due to ILD exacerbation and his serum IgG4 levels were elevated. Treatment with cyclosporine and prednisolone stabilized the ILD. However, 2 years later, he experienced a fever, myalgia, and mechanic’s hands, with high serum creatine kinase levels and muscle inflammation at MRI. The positivity of the PL-7 antibody was documented with an ELISA assay confirming the overlapping syndrome. The patient received two courses of steroid pulse treatments without improvement of the myopathy and combination therapy with IVIG with the relief of muscle involvement. Long-term outcome data are not reported [15].

Another instance of anti-synthetase/SD overlap syndrome was reported in January 2022 by Wang G. et al. [16] in a 71-year-old woman who presented with a one-month history of shortness of breath. She had experienced persistent dry mouth and gritty eyes for two years and was positive for ANA, anti-SS-A and anti-OJ antibodies. A HRCT scan showed non-specific interstitial pneumonia (NSIP), and pulmonary function tests suggested a moderate decrease in the diffusion function. A salivary gland biopsy confirmed focal lymphocytic sialadenitis, consistent with a diagnosis of SD. The patient had no evidence of other connective tissue diseases and was ultimately diagnosed as anti-synthetase/SD overlap syndrome. She was treated with methylprednisolone (20 mg/day), cyclophosphamide (0.4 g/week), and Tripterygium wilfordii (a traditional Chinese medicine) at a non-specified dosage. Her condition improved and she was discharged with a stable pulmonary function [16].

In November 2022, Mizuhashi Y. et al. [17] reported a sixth case of overlap syndrome in a 33-year-old woman who presented with a fever, muscle weakness, and exertional dyspnea. She received a diagnosis of anti-OJ antibody-positive polymyositis (PM), which overlapped with LES and SD. Treatment with prednisolone at 1 mg/kg/day led to significant symptom improvement; AZA was added after twelve months due to a muscle relapse with a good control of the disease activity and a tapering of the prednisolone dose [17].

Finally, in a large Japanese cohort study by Noguchi et al. (2017) [18] including 51 patients with ASyS, 3 patients were reported to have a concurrent diagnosis of SD. However, no clinical, serological, or imaging data were provided, preventing further the characterization of these overlap cases (not included in Table 1 due to insufficient data) [18].

## 5. Discussion

The differential diagnosis and treatment of ILD in the context of systemic rheumatic diseases is still challenging. Anti-Ro52/SSA positivity is associated with an increased frequency of ILD diagnosis in various autoimmune diseases [19]. Although anti-Ro52/SSA antibodies are more commonly considered diagnostic markers, emerging evidence suggests they may also contribute to the pathogenesis of ILD in connective tissue diseases, including SD, by interfering with TRIM21 regulatory pathways and promoting local immune-mediated tissue damage [20].

In clinical practice, a patient presenting with anti-Ro52/SSA antibody positivity, sicca symptoms, and ILD is typically interpreted as having SD, given its higher prevalence compared to ASyS. However, when ILD is rapidly progressive, or unresponsive to standard treatment, alternative diagnoses such as ASyS (or a potential overlap between the two conditions) should always be considered even in the absence of muscle involvement [21]. Notably, muscle involvement is not universally present in ASyS and, when present, may exhibit a milder phenotype characterized by isolated myalgia or the subclinical elevation of muscle enzymes, further complicating the diagnosis [22].

The case described in this report involved an anti-synthetase/SD overlap syndrome, differing in several respects from previously reported cases. The median age of those cases was 53 years, while our patient was 68. An advanced age may have contributed to the poor outcome, as it worsens the prognosis of systemic disease through a combination of a reduced physiological function, immunosenescence, comorbidities, complexities in pharmacological management, and frailty.

Three out of the six cases published in the literature showed overlap syndrome in which ASyS and SD were further associated with other diseases such as systemic lupus erythematosus, systemic sclerosis, or IgG4-related disease. Only three cases exhibited the same overlap condition of our patient, although some different serologic features were observed (Table 1). Notably, anti-PL-7 autoantibodies (also present in our patient) were observed in only one reported case of overlap involving ASyS, SD, and IgG4-related disease. That patient presented with predominant muscular involvement (completely absent in our patient) and a less severe form of usual interstitial pneumonia (UIP) compared to our patient.

All the patients with pulmonary involvement exhibited a moderate disease course regardless of the radiologic patterns. The most frequent manifestation of ILD, in the context of IIMs, is characterized by cryptogenic organizing pneumonia (COP) or a combination of COP and NSIP, which were the initial radiological findings documented in our patient (Figure 1) [23]. Nevertheless, the recurrent pulmonary infections observed in our patient progressively modified the radiologic ILD features (Figure 3), making the correct definition more challenging. Of note, no infections were described in the other cases and it remains unclear why our patient showed this important susceptibility to infections. All the reported patients underwent intensive immunosuppression treatment and no severe infectious complications were described in the published reports.

Lacking specific recommendations and a scientifically accepted standard of care for ILD in these forms, all the reported cases received different immunosuppressive regimens, making the comparability of these treatments on pulmonary damage progression difficult.

Five out of the six reported patients showed a predominant muscular involvement, responding favorably to immunosuppressive treatments with muscular function recovery. The other reported patient with an amyopathic form (as our patient), showed a better outcome and was discharged, whilst still being followed-up at the time of the data publication. The follow-up durations in the reported cases varied largely. Long-term observations were reported in two out of six cases (with follow-up times of 41 and 12 months), both exhibiting favorable outcomes, but with distinct antibody positivity and radiological characteristics compared to our patient.

ILD progression, associated with IIMs, exhibits extensive variability; thus, asymptomatic patients or patients experiencing a very low progression of lung involvement generally do not need any treatment, which is suggested for patients exhibiting a more severe degree of respiratory impairment, remarkable abnormalities in imaging or pulmonary function tests, and those who exhibit specific high-risk antibodies, such as anti-Ro/SSA, antimelanoma differentiation-associated gene 5 (MDA5), or antisynthetase antibodies [24].

Due to the rarity of the disease, no standardized treatment protocols or guidelines for those patients have been developed [25]. Several therapeutic approaches are currently used in clinical practice for treating ASyS, including AZA, methotrexate, tacrolimus, cyclophosphamide, cyclosporine, and mycophenolate mofetil, but none of them showed satisfactory effects in these patients [26].

In 2024, Kouranloo et al. [27] published the first systematic literature review and meta-analysis specifically focused on ASyS-associated ILD, including 514 patients from 10 studies. Immunosuppressive therapies, particularly cyclophosphamide and rituximab, were associated with significant improvements in lung function parameters. At 1-year, pooled data showed a mean increase in FVC of 14.1% and in DLCO of 15.1% compared to the baseline. Both treatments demonstrated effectiveness, with CYC showing a greater mean improvement in FVC (+17%) and DLCO (+6.3%) than RTX (+12.2% and +2.9%, respectively). However, no clear superiority of one regimen over the others could be established. The authors highlight the marked heterogeneity of treatment strategies, the lack of randomized controlled trials, and the importance of a multidisciplinary approach for optimal management [27].

Emerging evidence also points to the use of CD19-targeted CAR T cell therapy in ASyS patients with the disease refractory for conventional immunosuppressive treatments [28,29]. These preliminary findings may suggest a potential future therapeutic approach. However, further investigations are needed to evaluate the efficacy of specific immunosuppressive treatments in patients with these rare overlap syndromes.

Our patient initially benefited after immunosuppressive sequential therapy using Methylprednisolone and IVIG therapy, followed by micophenolate mofetil as a maintenance treatment protocol; nintendanib was subsequently chosen as the anti-fibrotic agent [30]. Despite an initial therapeutic response and partial disease remission, the clinical course was complicated by recurrent infectious episodes, likely due to cumulative immunosuppression, ultimately leading to the patient’s demise. This case highlights the inherent difficulty in managing rapidly progressive ILD, where the need for early and intensive immunosuppression must be carefully balanced against the risk of infectious complications, particularly in elderly or immunologically vulnerable patients.

## 6. Conclusions

The overlap syndrome described in this report may represent a significant clinical challenge, as it can present with atypical features that deviate from the classic inflammatory myopathy phenotype. It should be suspected in cases of rapidly progressive ILD, even in the absence of hallmark signs of myositis. Further investigations are needed to clarify whether anti-Ro52 antibodies, in combination with anti-synthetase antibodies, may contribute to the pathogenesis of ILD in an additive or synergistic manner.

Our case highlights the complex interplay among ASyS, SD, older age, and related frailty, which likely concurred to the poor outcome. Early diagnosis and treatment strategies focused on managing the underlying ILD, along with the careful management of age-related comorbidities, might improve prognosis in this specific subset of patients.

## Figures and Tables

**Table 1:** Summary of published cases of anti-synthetase/Sjögren overlap syndrome. Abbreviation: ANA, anti-nuclear antibodies; ASyS, anti-synthetase syndrome; SD, Sjögren disease; SLE, systemic lupus erythematosus; UIP, usual interstitial pneumonia; NSIP, non-specific interstitial pneumonia; CYC, cyclophosphamide; AZA, azathioprine; CyA, cyclosporin A.

Case Report	Age at Diagnosis	Gender	Overlap Syndrome	Antibodies	Clinical Presentation	ILD	Treatment	Outcome
Colafrancesco S. et al. (2015) [6]	42	female	- ASyS - SD	- ANA (pattern not reported) - anti Ro/SSA - anti La/SSB - anti JO1	- Raynaud Phenomenon: not reported - Arthralgia: not reported - Muscle weakness: yes - Muscle enzyme elevation: yes - Dyspnea: none	None	- Mycophenolate mofetil (2 g/die) - CyA (dosage not specified) - RTX 1 g × 2 doses, 2 weeks apart, every 6 months (5 cycles in total)	Excellent therapeutic response with sustained remission following RTX
	53	male	- ASyS - SD	- ANA (speckled and nucleolar 1/2560) - anti Ro/SSA - anti La/SSB - anti OJ	- Raynaud Phenomenon: not reported - Arthralgia: not reported - Muscle weakness: yes - Muscle enzyme elevation: yes - Dyspnea: yes	Not reported	- Pulse of steroids (dosage not specified) - Mycophenolate mofetil (2 g/die)	No data available on the long-term outcome
Hervier B. et al. (2011) [14]	21	female	- ASyS - Systemic sclerosis - SD	- ANA (speckled and nucleolar 1/2560) - anti Ro/SSA - anti La/SSB - anti OJ	- Raynaud Phenomenon: yes - Arthralgia: yes - Muscle weakness: yes - Muscle enzyme elevation: yes - Dyspnea: yes	UIP	- Prednisolone (1 mg/kg/day) - Six pulses of CYC 0.7 g/m2/monthly - AZA (2 mg/kg/day)	Improvements in muscular and pulmonary function (over 41 months of follow-up)
Yoshikawa M. et al. (2020) [15]	55	female	- ASyS - SD - IgG4-related disease	- anti Ro/SSA - anti PL-7 - anti OJ - high serum level of IgG4	- Raynaud Phenomenon: no - Arthralgia: no - Muscle weakness: yes - Muscle enzyme elevation: yes - Dyspnea: yes	UIP	- Prednisolone (60 mg/day) - CyA (125 mg/day) - Two courses of steroid pulse in combination with intravenous immunoglobuline on acute phase	Relief of muscle involvement (no data available on the long-term outcome)
Wang G. et al. (2022) [16]	71	female	- ASyS - SD	- ANA (1/320) - anti Ro/SSA - anti OJ	- Raynaud Phenomenon: yes - Arthralgia: no - Muscle weakness: no - Muscle enzyme elevation: not specified - Dyspnea: yes	NSIP	- Methylprednisolone 20 mg/die - CYC 0.4 g/week -Tripterygium wilfordii for 3 weeks	Improvements in pulmonary function (no data available on the long-term outcome)
Mizuhashi Y. et al. (2023) [17]	33	female	- ASyS - SD - SLE	- ANA (homogeneous 1:80, speckled 1:640, and cytoplasmic pattern positive) - anti ssDNA - anti Ro/SSA - anti La/SSB - anti OJ	- Raynaud Phenomenon: no - Arthralgia: no - Muscle weakness: yes - Muscle enzyme elevation: yes - Dyspnea: yes	bilateral diffuse cystic changes with diffuse interstitial pneumonia	- Prednisolone (1 mg/kg/day) - AZA (not specified dosage)	Improvement in muscles involvement (12 months of follow-up)
Our patient	68	female	- ASyS - SD	- ANA (anti-Golgi pattern 1:1280) - anti Ro/SSA - anti PL-7	- Raynaud Phenomenon: no - Arthralgia: no - Muscle weakness: no - Muscle enzyme elevation: no - Dyspnea: yes	NSIP	- Methylprednisolone bolus therapy (1 g/day for 3 days) - IVIG therapy (400 mg/Kg per day for 5 days) - Mycophenolate mofetil (3 g/day) - Nintedanib (300 mg/day)	The patient passed away 3 months after the initiation of immunosuppressive therapy

## Data Availability

The data presented in this study are available on request from the corresponding author. The data are not publicly available due to privacy and ethical restrictions.
